# Acute effects of 2.856 GHz and 1.5 GHz microwaves on spatial memory abilities and CREB-related pathways

**DOI:** 10.1038/s41598-021-91622-4

**Published:** 2021-06-11

**Authors:** Shengzhi Tan, Hui Wang, Xinping Xu, Li Zhao, Jing Zhang, Ji Dong, Binwei Yao, Haoyu Wang, Yanhui Hao, Hongmei Zhou, Yabing Gao, Ruiyun Peng

**Affiliations:** 1 PLA Strategic Support Force Characteristic Medical Center, Beijing, People’s Republic of China; 2 Department of Experimental Pathology, Beijing Institute of Radiation Medicine, Beijing, People’s Republic of China

**Keywords:** Hippocampus, Spatial memory, Synaptic plasticity

## Abstract

This study aimed to evaluate the acute effects of 2.856 GHz and 1.5 GHz microwaves on spatial memory and cAMP response element binding (CREB)-related pathways. A total of 120 male Wistar rats were divided into four groups: a control group (C); 2.856 GHz microwave exposure group (S group); 1.5 GHz microwave exposure group (L group); and 2.856 and 1.5 GHz cumulative exposure group (SL group). Decreases in spatial memory abilities, changes in EEG, structural injuries, and the downregulation of phosphorylated-Ak strain transforming (p-AKT), phosphorylated-calcium/calmodulin-dependent protein kinase II (p-CaMKII), phosphorylated extracellular signal regulated kinase (p-ERK) and p-CREB was observed 6 h after microwave exposure. Significant differences in the expression of p-CaMKII were found between the S and L groups. The power amplitudes of the EEG waves (θ, δ), levels of structural injuries and the expression of p-AKT, p-CaMK II, p-CREB, and p-ERK1/2 were significantly different in the S and L groups compared to the SL group. Interaction effects between the 2.856 and 1.5 GHz microwaves were found in the EEG and p-CREB changes. Our findings indicated that 2.856 GHz and 1.5 GHz microwave exposure induced a decline in spatial memory, which might be related to p-AKT, p-CaMK II, p-CREB and p-ERK1/2.

## Introduction

The brain is one of the most sensitive organs to electromagnetic radiation^1–3^. The effects of microwave radiation on spatial learning and memory ability have been reported in many studies^4,5^, but the mechanisms are not clear. Most studies focused on the biological effects of microwaves at a single frequency. Microwaves with frequencies of 2.856 GHz and 1.5 GHz belong to the S and C bands, respectively, and are widely used in telecommunication, especially radar communication. Microwaves with different frequencies have different electromagnetic properties, which might induce different biological effects. People who operated radars or lived near a radar station are generally exposed to a complex electromagnetic environment of different frequencies. Therefore, in this study, we used two different frequencies (2.856 GHz and 1.5 GHz) of microwaves with the same power density (10 mW/cm^2^) to determine the properties and accumulative biological effects of the two different microwaves.

The Morris water maze (MWM), as a classic test for spatial learning and memory in rodents, has been widely used to assess microwave radiation-induced impairment of spatial memory^6–9^. Electroencephalographic (EEG) data are frequently used to assess the effects of microwave exposure on brain bioelectrical activity because of the sensitivity of these scans to immediate changes in neural processes^10–12^. However, previous studies paid little attention to the acute effects of microwave radiation. Therefore, the present study evaluated the acute and accumulative effects of two different microwave frequencies on learning and memory abilities and bioelectrical activity.

Microstructural observations should not be neglected in investigations of the possible mechanisms. Neurons are the basis for signal transmission and learning and memory development. Microwave radiation induces neural cell apoptosis via the classic mitochondrion-dependent caspase-3 pathway^13^. The hippocampus and cortex are important brain areas for learning and memory abilities. Therefore, it is necessary to observe changes in the neurons in the hippocampus and cortex, especially the occurrence of neuronal apoptosis.

Many studies have shown that cAMP-response element binding protein (CREB) plays an important role in learning and memory processes^14–16^. CREB is a key transcription factor that regulates the expression and activity of genes to alter the function of neurons^17–19^. CREB is also a connection between several neural function-related pathways. The phosphoinositide 3-kinase (PI3K)/Ak strain transforming (AKT) pathway, calcium/calmodulin-dependent protein kinase II (Ca^2+^/CaMKII) pathway and mitogen-activated protein kinase (MEK)/extracellular signal regulated kinase (ERK) pathway are three key upstream pathways of CREB^20–22^ and regulate the expression of multiple downstream functional proteins in the nervous system. Activation of the PI3K/AKT pathway was observed in neuron-like cells after microwave exposure, and the changes were considered protective responses^23–25^. The influx of calcium increases after microwave radiation in neuron-like cells^26^, which suggests that the Ca^2+^/CaMKII pathway participates in microwave bioeffects. Increased ERK phosphorylation is detected in response to extremely low-frequency electromagnetic fields^27^. However, the relationship between these microwave-sensitive pathways is not clear.

The present study detected the expression levels of AKT, p-AKT, CaMK II, p-CaMK II, CREB, p-CREB, ERK1/2 and p-ERK1/2. We compared the molecular mechanisms between the different frequency microwaves and explored the possible interaction effects between the two frequencies. These findings could contribute to our understanding of the mechanisms of microwave-induced spatial memory impairment. Two different microwave frequencies were considered two different factors. Therefore, a 2 × 2 factorial design was used to evaluate whether an interaction occurred between the frequencies.

## Materials and methods

### Animals and groups

The Ethics Committee of the Academy of Military Medical Science approved all experimental protocols for animal care, handling and experimentation (IACUC-DWZX-2020–0780), and all experiments were performed in accordance with relevant guidelines and regulations. All the methods were carried out in accordance with the Guide for the Care and Use of Laboratory Animals published by the US National Institutes of Health (NIH Publication No. 85–23, revised 1996). The study was carried out in compliance with the ARRIVE guidelines (http://www.nc3rs.org.uk/page.asp?id=1357).

A total of 120 male Wistar rats (weights 200 ± 20 g) (n = 30 per group) were obtained from the Laboratory Animal Center of the Beijing Institute of Radiation Medicine and housed in a specific pathogen-free (SPF)-grade animal facility. All experiments were performed between 8:00 and 15:00.

The 2.856 GHz and 1.5 GHz microwaves lie within the S band and L band, respectively. Therefore, we used the letter “S” to represent the 2.856 GHz exposure group and the letter “L” to represent the 1.5 GHz exposure group. “SL” represented the 2.856 and 1.5 GHz cumulative microwave exposure group. All rats were randomly divided into four groups: (1) the control group (C); (2) the 2.856 GHz microwave exposure group (S); (3) the 1.5 GHz microwave exposure group (L); and (4) the 2.856 and 1.5 GHz cumulative microwave exposure (SL).

### Microwave exposure and dosimetry

Two microwave sources with a frequency of 2.856 GHz or 1.5 GHz were placed next to each other in an electromagnetic shield chamber. The exposure procedures were the same as those used by Tan^28^. Briefly, rats in the S and L groups were exposed to 2.856 GHz or 1.5 GHz microwaves, respectively, with an average power density of 10 mW/cm^2^ for 6 min. Rats in the SL group were first exposed to the 2.856 GHz antenna (the S band) for 6 min then moved parallel to the 1.5 GHz antenna (the L band) for 6 min via a conveyor belt. The interval time between the two exposure procedures was very short and may be negligible.

The specific absorption rate (SAR) values were calculated as described in our previous paper^2^. The SAR values were calculated as described in our previous paper^28^. The SAR values of the whole body for C, S and L were 0, 3.3 and 3.7 W/kg, respectively. For the SL group, the SAR values of the whole body were 3.3 W/kg for the first 6 min and 3.7 W/kg for the last 6 min.

### Morris water maze

The procedures for the Morris water maze strictly followed those of previous reports^6,29^. Briefly, a circular pool with a diameter of 150 cm was filled with water at 23 ± 0.5 °C in a room with constant brightness and divided into four equal quadrants. A platform with a diameter of 12 cm and a height of 15 cm was submerged 1.5 cm under the water surface. A Morris water maze video analysis system (Beijing Sunny Instrument Co., Ltd., Beijing, China) based on moving object detection and tracking was used.

Sixty male Wistar rats (n = 15 per group) in a fixed order were trained to find the submerged platform in 4 trials before microwave exposure. The training experiment lasted 2 days. The rats were trained to find the submerged escape platform for three consecutive days before the initiation of radiation. Each trial consisted of four trials, which started from four different starting positions. These positions were equally located around the perimeter of the pool and used in a fixed order. Each trial had a maximum duration of 60 s. Rats that did not find the platform within 60 s in the training tests were placed on the platform for 15 s. The rats were exposed to microwave radiation on day 3. Navigation tests were performed 6 h after microwave exposure to detect acute changes in spatial memory. Four trials were conducted, and each trial had a maximum duration of 60 s. The duration of each rat to find the platform was recorded separately, and the average escape latency (AEL) of each rat in the water maze was obtained by calculating the average of 4 trials for each experimental animal for each training day. The behavior of the rats was digitally recorded by using a SLY-MW system (Beijing Sunny Instrument Co., China), and the AEL was analyzed.

### EEG recordings

Twenty male Wistar rats (n = 5 per group) were anesthetized with 1% sodium pentobarbital (0.5 ml/100 g). An Mp-150 multichannel physiological recording and analysis system (Biopac Company, USA) was used. The electrodes were connected to the amplifier, and the rats were recorded for 3 min in a quiet state 6 h after microwave exposure. Afterward, power spectral analyses were performed.

### Hematoxylin and eosin (H&E) and TUNEL staining of the hippocampus

Twenty male Wistar rats (n = 5 per group) were anesthetized with sodium pentobarbital (50 mg/kg, IP) 6 h after microwave exposure. The skin was removed from the head of the anesthetized rats. The bregma point of the brain tissue was marked and drilled, and the brain tissue was separated. The right brain tissue was cut 3.3–3.8 mm behind the bregma point. The brain tissues were fixed in formalin. The right brains were separated and fixed in a 10% buffered formalin solution for at least 1 week. Coronal brain sections, including the hippocampus, were made. The sections were selected at 3.6 mm behind the bregma point. The sections were dried, dewaxed, dipped in hematoxylin for 15 min and washed for 20 min with tap water. Eosin was used to stain the sections for 15 s. Sections were dehydrated in an alcohol gradient and cleared in xylene. Cover slips were placed on the slides, and the stained sections were observed under a Leica DM6000 light microscope (Leica, Germany). The DG area of the hippocampus was observed with a light microscope.

Paraffin sections of the hippocampus were prepared 6 h after microwave exposure. The sections were dried, dewaxed, and treated with proteinase K (20 μg/ml, Roche, Switzerland) for 10 min to make the cell membrane permeable. The sections were then treated with the reaction solution mixture (enzyme solution and label solution, Roche, Switzerland) for the TdT-mediated dUTP nick-end labeling (TUNEL) reaction. After treatment with biotin-labeled horseradish peroxidase (HRP, ZSGB, Beijing, China), 3,3′-diaminobenzidine (DAB) chromogen (ZSGB, Beijing, China) was used to develop the color. A Leica DM6000 light microscope was used to observe the stained sections. The DG areas were selected at 3.6 mm posterior to bregma, 2–2.5 mm lateral to the mid-line and at a depth of approximately 3.5 mm.

Pathological analyses used five rats from each group. Ten sections of each rats were made. Approximately 60 cells in the DG area were selected from each section. The mean optical density (MOD) in H&E sections referred to the ratio of the area of the dark blue nucleus to the total area of the nucleus in the observed cells. The integrated optical density (IOD) referred to the whole optical density of all positive nuclei from observed cells in the TUNEL-stained sections. As for calculating IOD, the background from each section was subtracted and each rat was used for one datapoint.

### Western blotting

Twenty male Wistar rats (n = 5 per group) were anesthetized, and the entire left hippocampus was separated. The tissue was homogenized for Western blotting (WB). The expression levels of p-AKT, p-CaMKII, p-CREB, p-ERK1/2, AKT, CaMKII, CREB and ERK1/2 in the hippocampi of the rats were detected by WB 6 h after microwave exposure. Protein extraction and quantification and WB procedures were performed according to Xiong’s research^26^. Primary antibodies against p-AKT (Abcam, UK), p-CaMKII (Abcam, UK), p-CREB (Abcam, UK), p-ERK1/2 (Abcam, UK), AKT (Abcam, UK), CaMKII (Abcam, UK), CREB (Abcam, UK) and ERK1/2 (Abcam, UK) were used at 1:1000 dilutions. The dark room film development method was used to obtain images of the protein bands, which were quantified using ImageJ 1.80 software. An HP LaserJet pro MFP m126a scanner was used for film scanning, and the scanned images were analyzed via Microsoft Word software for simple color saturation and overall brightness adjustment of the image. The protein abundance was normalized to that of GAPDH.

### Statistical analysis

The data are shown as the means and standard deviation ($\overline{X }$±*S*). The interaction effects were analyzed by a 2 × 2 factorial design. The 2.856 GHz microwave exposure and 1.5 GHz microwave exposure were regarded as two factors in the factorial design. Two-way ANOVA and multiple comparisons were performed with SPSS 19.0 software, and ^Δ^*p* < 0.05 or ^ΔΔ^*p* < 0.01 were considered significant interaction effects. If interaction effects were observed, then a regression analysis was used to determine which factor played a more important role. Factors with larger absolute values of the standardized regression coefficient (β value) were considered to be of greater importance. If there were significant main effects in the anova, the posthoc multiple comparisions were conducted by the bonferroni analysis, with *p < 0.05 or **p < 0.01 (*vs.* C), ^•^*p* < 0.05 or ^••^*p* < 0.05 (*vs.* SL), ^#^*p* < 0.05 or ^##^*p* < 0.01 (L *vs.* S).

## Results

### Acute impairment of spatial memory abilities after 2.856 GHz and 1.5 GHz microwave exposure

The average escape latency (AEL) indicates the time needed for the rats to find the platform in the MWM. A prolonged AEL indicates a decline in spatial memory abilities.

**Figure 1:**
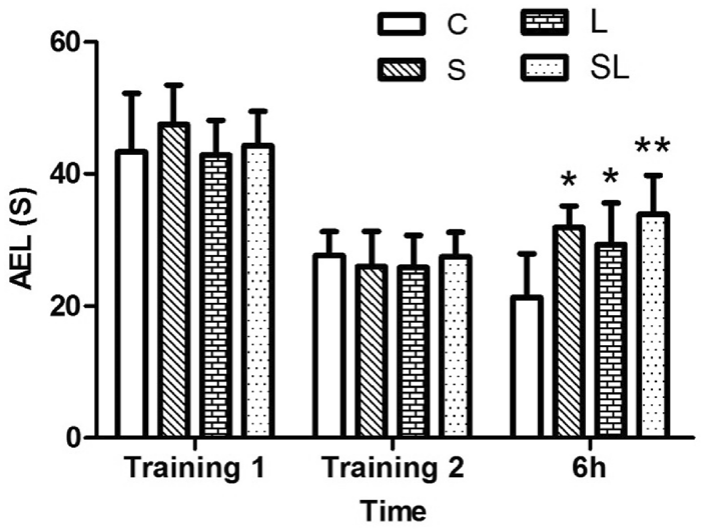
The AELs of the rats during the MWM test. The data are presented as the means ± SDs. *p < 0.05 and **p < 0.01. compared to the C group. “C” stands for the control group, “S” stands for the 2.856 GHz microwave exposure group, “L” stands for the 1.5 GHz microwave exposure group, and “SL” stands for the 2.856 and 1.5 GHz cumulative exposure group.

After two days of training before microwave exposure, there were no differences in AEL between the C, S, L and SL groups, which indicated the same basic learning and memory abilities of the four groups before microwave exposure. Compared to that in the C group, the AEL of the rats in the S (*p* = 0.012), L (*p* = 0.049) and SL groups (*p* = 0.001) significantly increased 6 h after microwave exposure (Fig. 1).

No significant differences were found between the S and L groups.

No significant differences were found between the single-exposure groups (S, L) and the cumulative group (SL).

Factorial analysis of the four groups (C, S, L and SL) revealed no significant interaction effects between the 2.856 GHz and 1.5 GHz microwave radiation (*p* = 0.273). According to the standardized regression coefficient (β value) of the regression analysis, 2.856 GHz microwave radiation played major roles in the changes in AEL(β_S_ = 0.511, β_L_ = 0.335,|β_S_| >|β_L_|).

### Acute inhibition of EEG after 2.856 GHz and 1.5 GHz microwave exposure

**Figure 2:**
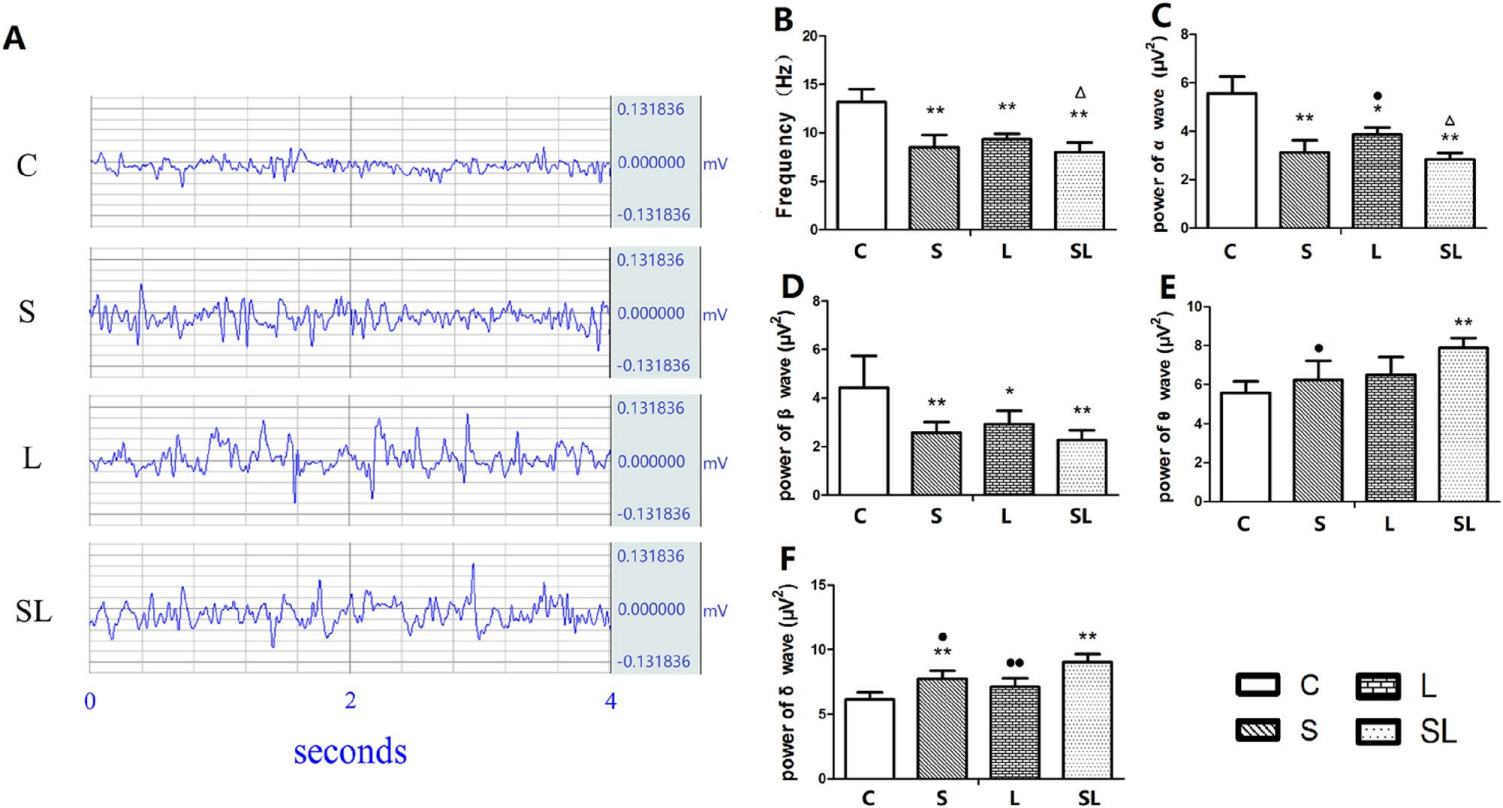
Changes in the frequency and power of α, β, θ and δ waves in the EEGs. The data are presented as the means ± SDs. (**A**) shows the waveforms of the EEGs in the four groups. (**B**) shows the statistical results of the EEG frequency. (C)-(F) show the statistical results of the powers of α, β, θ and δ waves, respectively. *p < 0.05 and **p < 0.01 compared to the C group. 1. ^•^*p* < 0.05 and ^••^*p* < 0.01 compared to the cumulative group. ^Δ^*p* < 0.05 indicates the existence of interaction effects between the 2.856 GHz and 1.5 GHz microwaves. “C” stands for the control group, “S” stands for the 2.856 GHz microwave exposure group, “L” stands for the 1.5 GHz microwave exposure group, and “SL” stands for the 2.856 and 1.5 GHz cumulative exposure group.

EEG examinations were performed 6 h after microwave exposure, and the EEG waveform is shown in Fig. 2A. The frequency of the EEG decreased significantly in the S (*p* = 0.000), L (*p* = 0.001) and SL (*p* = 0.000) groups (*p* < 0.01) compared with the C group (Fig. 2B). The power amplitudes of the α waves significantly decreased in the S (*p* = 0.000), L (*p* = 0.002) and SL (*p* = 0.000) groups compared with the C group, and the power amplitudes of the β waves significantly decreased in the S (*p* = 0.019), L (*p* = 0.046) and SL (*p* = 0.009) groups compared with the C group (Fig. 2C,D). The power amplitudes of the θ waves in the SL (*p* = 0.007) group and δ waves in the S (*p* = 0.008) and SL (*p* = 0.000) groups significantly increased compared with those in the C group (Fig. 2E,F).

No significant changes were found between the S and L groups.

The power amplitude of the α wave significantly increased in the L group (*p* = 0.045) compared with the SL group (Fig. 2B). The power amplitude of the θ wave in the S group was lower than that in the SL group (*p* = 0.032) (Fig. 2E). The power amplitudes of the δ waves significantly decreased in the S (*p* = 0.023) and L (*p* = 0.004) groups compared with the SL group (Fig. 2F).

Factorial analysis of the four groups (C, S, L and SL) revealed significant interaction effects between the 2.856 GHz and 1.5 GHz microwaves for the frequency changes in the EEG (*p* = 0.018) and the power amplitude changes in the α wave (*p* = 0.031). According to the standardized regression coefficient (β value) of the regression analysis, 2.856 GHz microwave radiation played major roles in the changes in EEG frequency (β_S_ =  − 0.69, β_L_ =  − 0.458, |β_S_| >|β_L_|) and the power of the α wave (β_S_ =  − 0.747, β_L_ =  − 0.435, |β_S_| >|β_L_|). These results indicated that 2.856 GHz microwave radiation played major roles in the changes in the EEG for the cumulative exposure group.

### Exposure to 2.856 GHz and 1.5 GHz microwaves caused significant structural injuries in the hippocampus

**Figure 3:**
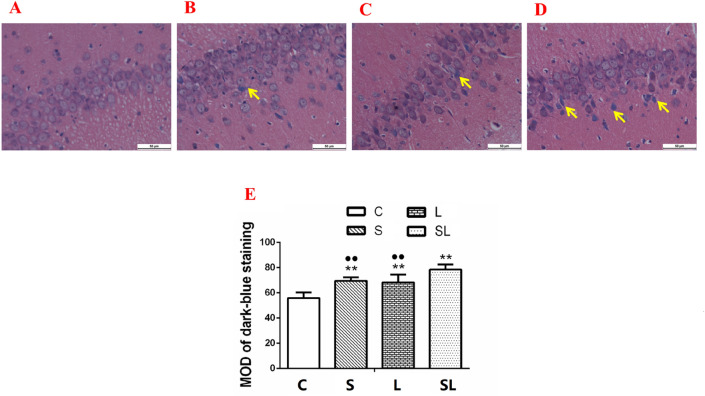
Pathological injuries in the hippocampus of rats 6 h after 2.856 GHz and 1.5 GHz microwave exposure. The microstructures were detected by LM 6 h after exposure to support the behavioral results. (**A**–**D**) Hippocampi of the C group, S group, L group and SL group. (E) Statistical analysis results of the MOD of dark blue staining in hippocampi. Compared to the C group, **p < 0.01. Compared to the cumulative group, ^••^*p* < 0.01. There was no neuron degeneration detected by A and E., and injuries that included karyopyknosis, an irregular arrangement of cells, cell edema, and a broadening pericellular space were found in (**B**–**D**). Most injuries occurred in (**D**). (H&E, scale bar = 50 μm). “C” stands for the control group, “S” stands for the 2.856 GHz microwave exposure group, “L” stands for the 1.5 GHz microwave exposure group, and “SL” stands for the 2.856 and 1.5 GHz cumulative exposure group. The data are presented as the means ± SDs.

To assess morphological changes in neurons, histological examinations of the hippocampus were carried out. Obvious injuries in the DG areas of the hippocampus were found in the microwave exposure groups (S, L and SL) compared with the C group (Fig. 3A–D). The injuries included karyopyknosis and cell edema, as shown by the dark blue-stained nuclei. Therefore, we analyzed the dark blue areas using statistical methods. The MOD of the dark blue area of the nuclei increased significantly in the hippocampus in the S (*p* = 0.000), L (*p* = 0.000) and SL (*p* = 0.000) groups compared with the C group (Fig. 3E). The MOD of the dark-blue areas of the hippocampus decreased significantly in the S (*p* = 0.004) and L (*p* = 0.003) groups compared with the SL group, which indicated that the most significant injuries occurred in the cumulative group (Fig. 3E). There were no significant interactions after the factorial analysis. According to the standardized regression coefficient (β value) of the regression analysis, 2.856 GHz microwave radiation played major roles in the changes in MOD of dark blue-stained nuclei of hippocampus (β_S_ = 0.729, β_L_ = 0.621, |β_S_| >|β_L_|).

### Exposure to 2.856 GHz and 1.5 GHz microwaves induced cell apoptosis in the hippocampus

**Figure 4:**
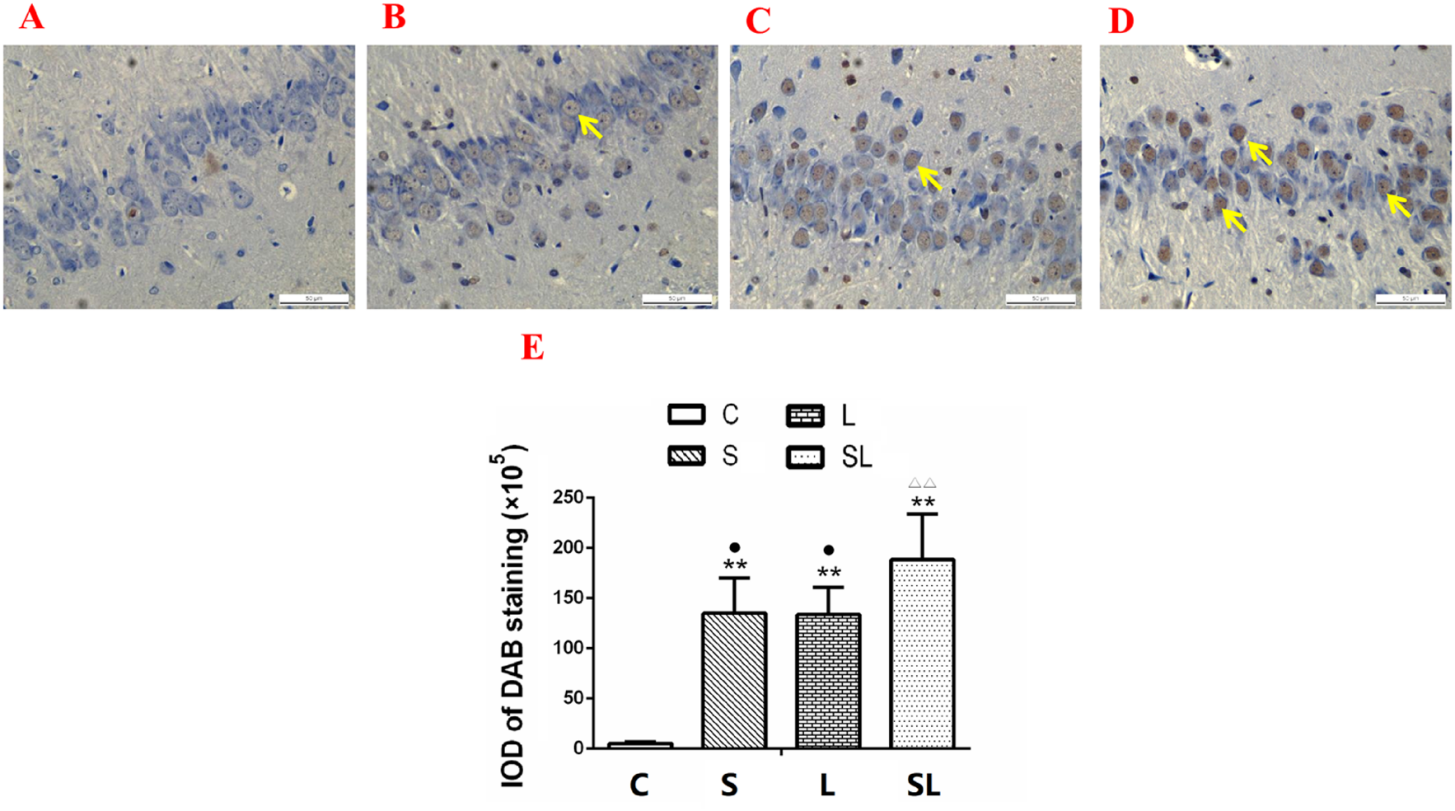
Cell apoptosis in the hippocampus of rats 6 h after 2.856 GHz and 1.5 GHz microwave exposures, as detected by TUNEL staining. (**A**–**D**) Hippocampi of the C group, S group, L group and SL group (TUNEL, scale bar = 50 μm). (E) IOD changes in the hippocampus. Compared to the C group, **p < 0.01. Compared to the cumulative group, ^•^*p* < 0.05. ^ΔΔ^*p* < 0.01 indicates the existence of interaction effects between the 2.856 GHz and 1.5 GHz microwaves. “C” stands for the control group, “S” stands for the 2.856 GHz microwave exposure group, “L” stands for the 1.5 GHz microwave exposure group, and “SL” stands for the 2.856 and 1.5 GHz cumulative exposure group. The data are presented as the means ± SDs.

To evaluate changes in neuronal cells, apoptosis was analyzed by TUNEL staining 6 h after microwave exposure. Deep-dyed nuclei were observed in the DG area of the hippocampi in the S, L and SL groups, which indicates the occurrence of apoptosis in neurons (Fig. 4A–D). The integrated optical density (IOD) of DAB staining in the hippocampus increased significantly in the S (*p* = 0.000), L (*p* = 0.000) and SL (*p* = 0.000) groups compared with the C group (Fig. 4E). The IOD of DAB staining in the hippocampus decreased significantly in the S (*p* = 0.022) and L (*p* = 0.013) groups compared with the SL group (Fig. 4E). Factorial analysis revealed significant interaction effects in the hippocampus and cortex between the 2.856 GHz microwave radiation and 1.5 GHz microwave radiation (*p* = 0.002) groups (Fig. 4E). According to the standardized regression coefficients (β value) of the regression analysis, 1.5 GHz microwave radiation played major roles in IOD value changes in the hippocampus ((β_S_ =  − 0.61, β_L_ =  − 0.666, |β_S_| <|β_L_|) and (β_S_ = 0.544, β_L_ = 0.603, |β_S_| <|β_L_|), respectively) (Fig. 4E). These results indicated that cell apoptosis occurred in the hippocampus and that 1.5 GHz microwave exposure played major roles in the changes in the cumulative exposure group.

### Changes in CREB-related signaling proteins after 2.856 GHz and 1.5 GHz microwave exposure

**Figure 5:**
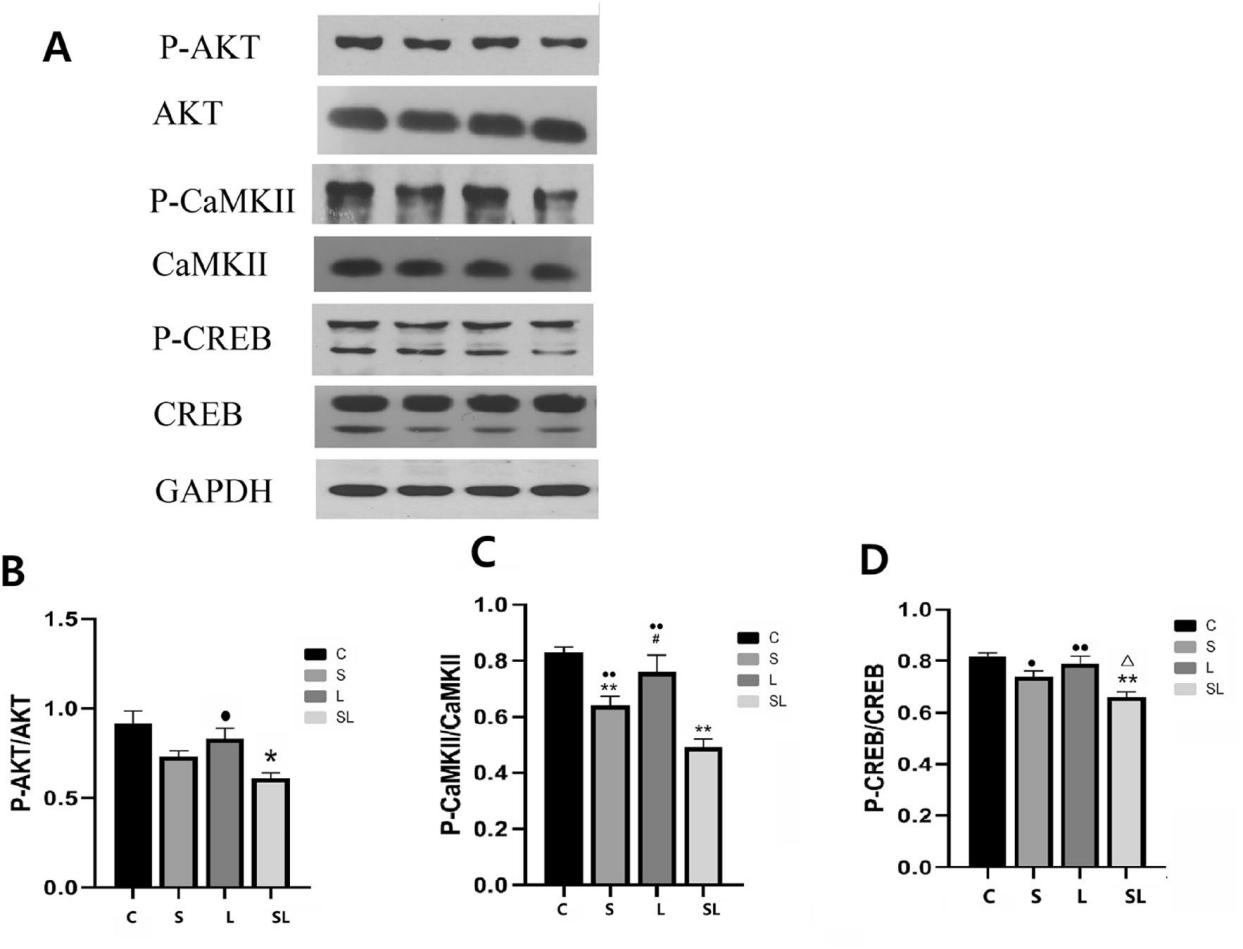
Comparisons of the levels of p-AKT (**B**), p-CaMKII (**C**), and p-CREB (**D**) in the hippocampi of the C, S, L and SL groups. (**A**) Expression of four proteins in the C, S, L and SL groups. (**B**) Statistical analysis results of p-AKT in the four groups. (**C**) Statistical analysis results of p-CaMKII in the four groups. (**D**) Statistical analysis results of p-CREB in the four groups. The data are presented as the means ± SDs. *p < 0.05 and **p < 0.01 compared to the C group. ^#^*p* < 0.05 compared to the S10 group. ^•^*p* < 0.05 and ^••^*p* < 0.01 compared to the cumulative group. ^Δ^*p* < 0.05 indicates the existence of interaction effects between the 2.856 GHz and 1.5 GHz microwaves. “C” stands for the control group, “S” stands for the 2.856 GHz microwave exposure group, “L” stands for the 1.5 GHz microwave exposure group, and “SL” stands for the 2.856 and 1.5 GHz cumulative exposure group.

**Figure 6:**
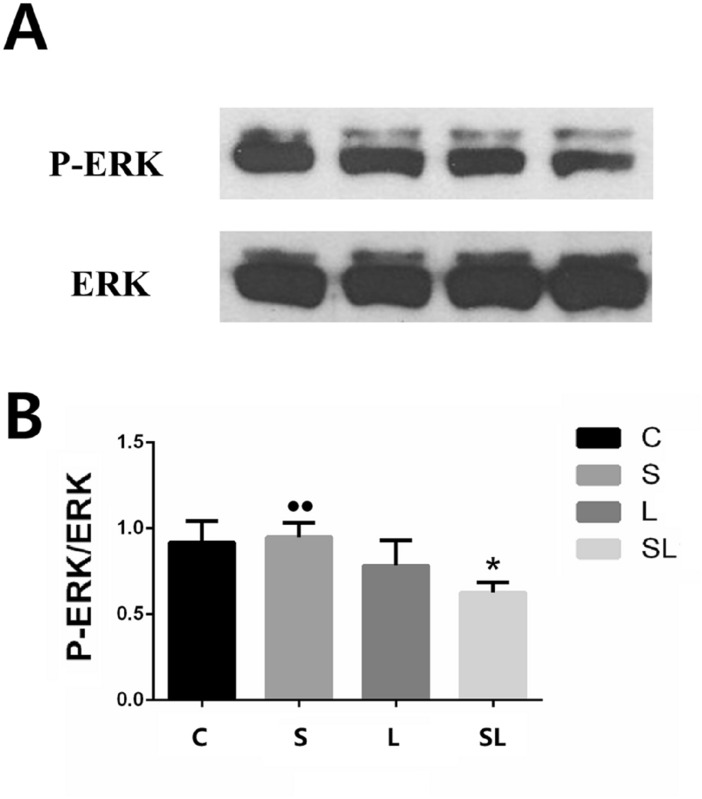
Comparison of the levels of p-ERK and ERK in the hippocampi of the C, S, L and SL groups. (**A**) Expression of four p-ERK and ERK proteins in the C, S, L and SL groups. (**B**) Statistical analysis results of p-ERK/ERK. The data are presented as the means ± SDs.*p < 0.05. ^••^*p* < 0.01 compared to the cumulative group. “C” stands for the control group, “S” stands for the 2.856 GHz microwave exposure group, “L” stands for the 1.5 GHz microwave exposure group, and “SL” stands for the 2.856 and 1.5 GHz cumulative exposure group.

Six hours after microwave exposure, p-AKT/AKT (*p* = 0.015), p-CREB/CREB (*p* = 0.001) (Fig. 5A,B,D) and p-ERK/ERK (*p* = 0.011) (Fig. 6A,B) decreased significantly in the SL group, and the expression of p-CaMKII/CaMKII decreased significantly decreased in the S (*p* = 0.009) and SL (*p* = 0.000) groups (Fig. 5A,C) compared with the C group.

Significant differences were also found between the S and L groups. The expression of p-CaMKII/CaMKII increased significantly in the L group (*p* = 0.021) compared with the S group.

The expression of p-AKT/AKT increased significantly in the L group compared with the SL group (*p* = 0.024). The expression levels of p-CaMKII/CaMKII increased significantly in the S (*p* = 0.006) and L (*p* = 0.000) groups compared with the SL group (Fig. 5A–C). The expression levels of p-CREB/CREB increased significantly in the S (*p* = 0.013) and L (*p* = 0.001) groups compared with the SL group (Fig. 5A,D). The expression of p-ERK increased significantly in the S group (*p* = 0.007) compared with the SL group (Fig. 6A,B).

Factorial analysis of the four groups (C, S, L and SL) revealed significant interaction effects between the 2.856 GHz and 1.5 GHz microwaves only for p-CREB/CREB (*p* = 0.046) expression. According to the standardized regression coefficient (β value) of the regression analysis, the 2.856 GHz microwaves played major roles in the decreased expression of p-AKT/AKT (β_S_ = -− 8.33, β_L_ =  − 0.417, |β_S_| >|β_L_|), p-CaMKII/CaMKII (β_S_ =  − 8.845, β_L_ =  − 0.396, |β_S_| >|β_L_|) and p-CREB/CREB (β_S_ =  − 0.757, β_L_ =  − 0.326, |β_S_| >|β_L_|) (Fig. 5). According to the standardized regression coefficient (β value) of the regression analysis, the 1.5 GHz microwaves played major roles in the decreased expression of p-ERK/ERK (β_S_ =  − 0.199, β_L_ =  − 0.751, |β_S_| <|β_L_|) (Fig. 6) .

## Discussion and conclusions

Most previous studies focused on the dose-dependent biological effects of a single-frequency microwave exposure^26,30,31^. However, with the increasingly serious microwave pollution in our living environments, people are exposed to microwaves at multiple frequencies. The energy distributions of microwaves with different frequencies in the organisms are different, and the biological effects depend on the energy distribution. Therefore, we hypothesized that the responses of organisms to microwaves of different frequencies would be different.

The cumulative group received more power than the single-frequency-exposure groups. Previous studies found dose-dependent effects of microwaves and showed that higher microwave power could lead to more severe effects^2,28^. The effects in the cumulative exposure group (SL) were more serious than those in the single-frequency-exposure groups (S, L) in the EEG, structural injuries, and the expression of p-AKT/AKT, p-CaMKII/CaMKII, p-CREB/CREB and p-ERK/ERK, which might be due to the larger dose exposure.

The MWM test is a classic method for detecting spatial learning and memory abilities^32,33^. Long-term exposure to 2.856 GHz microwaves prolonged the AELs in rats in a previous study^5^, but this study examined only the effects of one microwave frequency. We also found prolonged microwave-induced AELs. We also compared the effects between the 2.856 GHz and 1.5 GHz microwaves and found no differences in the AELs, which demonstrated that 2.856 GHz and 1.5 GHz could lead to decreased spatial memory abilities in Wistar rats. No differences were found between the cumulative exposure group and single-frequency-exposure groups, and no interaction effects were found by the factorial analysis of the AELs.

EEG scans directly reflect the physiological activities of the brain and are commonly used to evaluate brain function^34,35^. Changes in EEG activity results in many degenerative brain diseases^36^. EEG also plays important roles in the neuromodulatory balance of memory and mental illness^37^. Thuroczy reported that local brain exposure to 4 GHz continuous waves (CW) increased the power of δ waves^38^. Four kinds of waves, α (12–30 Hz), β (8–12 Hz), θ (4–8 Hz) and δ (1–4 Hz), lie within the EEG spectrum. The α and β waves appear in a relaxed or nervous status of the brain, and the θ and δ waves appear in a tired or sleepy state of the brain^39^. Our results indicated that microwave exposure caused rats to enter a more tired or depressed state.

Many studies found that microwave radiation induced structural damage in brain tissue^11,40^. The present study found tissue injuries in the 2.856 GHz and 1.5 GHz microwave exposure groups. The injuries were similar between the two single-frequency-exposure groups. The most serious injuries were observed in the cumulative exposure groups. To clarify the type of injuries, TUNEL staining was conducted. Cell apoptosis was observed in the hippocampus and cortex of the exposure groups. Previous studies found that neuroapoptosis in developing brains might cause long-term learning and memory abnormalities^41^. Apoptosis might explain the microwave-induced learning and memory impairments.

Analyses of the MWM, EEG, H&E staining and TUNEL staining revealed no significant differences between the 2.856 GHz microwave exposure and 1.5 GHz microwave exposure, which suggested that microwaves with frequencies in similar ranges induced similar effects. Accumulative microwave exposure induced serious changes. Therefore, dose accumulation played a more important role than single frequency microwave exposure. Notably, interaction effects were found for the changes in the EEG frequency, power amplitudes of α waves and IOD values of the TUNEL staining in the hippocampus. The standardized regression coefficients showed that the major factors involved in the functional and structural changes were different. For the MWM, EEG changes and p-AKT, p-CaMKII and p-CREB, The 2.856 GHz microwaves played major roles for the EEG changes, and 1.5 GHz microwave exposure played a major role in the TUNEL changes and p-ERK. The results indicated that the mechanisms of accumulative microwave exposure were complicated. The 2.856 GHz and 1.5 GHz microwaves may play different roles in the functional and structural changes of the accumulative exposure. The reasons for this result might be related to the energy and penetration depth of the microwaves.

CREB is an important transcription factor that plays an important role in the processes of learning and memory^42–44^. The phosphorylation of CREB affects the expression or activation of genes and the overall behavior of neurons^45^. Long-term facilitation alters CREB phosphorylation by increasing cAMP, which leads to the expression of related genes and ultimately results in changes in functional protein expression^46^. Multiple signaling pathways, such as the PI3K/AKT pathway^21^, Ca^2+^/CaMKII pathway^47^ and MEK/ERK pathway^20^ regulate the phosphorylation of CREB. In this study, the key signaling molecules in these signaling pathways were detected. We found that the phosphorylation levels of key signaling molecules, such as p-AKT/AKT, p-CaMK II/CaMKII, p-CREB/CREB and p-ERK/ERK, were downregulated after microwave radiation.

The levels of p-AKT, p-CaMK II and p-ERK are closely related to synaptic functions. Lee et al*.* reported that activation of the PI3K/Akt/mTOR pathway promoted dendritic spine formation and excitatory synapse development in hippocampal neurons^48,49^. Zhao et al*.*^50^ found that decreased AKT phosphorylation contributed to hippocampal neuronal apoptosis in rats. The decreased p-AKT level after microwave exposure in the present study might be related to microwave-induced neuronal apoptosis. Mohajerani et al. found that the activated MAPK/ERK pathway led to transcriptional regulation and new protein synthesis in postsynaptic neurons^51^. Curtis et al. found that genes regulated by CREB or other transcription factor targets of the CaMK and ERK pathways mediated important adaptive responses to changes in synaptic activity, such as changes in synaptic strength and the regulation of neuronal survival and death^52^. Therefore, we concluded that the decreased p-AKT/AKT, p-CaMKII/CaMKII and p-ERK/ERK levels resulted in a decrease in synaptic function and learning and memory functions. The decreased p-CREB/CREB levels might be the result of the inhibition of these three pathways.

In terms of the molecular level, differences in the expression of p-CaMKII/CaMKII between 2.856 GHz and 1.5 GHz were found, which suggests the different sensitivities of the signal pathways to microwaves with different frequencies. Interaction effects of the two microwave frequencies were found for the changes in p-CREB/CREB. The accumulative exposure of the 2.856 GHz and 1.5 GHz microwaves aggravated the downregulation of p-CREB/CREB. Previous studies used combined exposure to communication microwaves (849 MHz and 1.95 GHz), but none of these studies analyzed the interaction effects^53–56^.

The present study provides new insights into the biological effects of microwave radiation. Microwaves affect multiple metabolic pathways, and the frequency played an important role in the biological effects. The results related to frequency-dependent effects suggested that microwave safety standards should be based on the frequency. Past safety standards were primarily based on radiation power, and radiation frequency was defined only in a rough range^57^. The interaction effects suggested that the biological effects caused by electromagnetic waves were much more complicated than previously thought. More attention should be given to this field.
